# Sex-Specific Determinants of Physical Activity Among Korean Adults Using Weight-Control Medications: A Cross-Sectional Study

**DOI:** 10.3390/healthcare14162570

**Published:** 2026-08-17

**Authors:** Bo-Young Kim

**Affiliations:** Department of Health and Medical Administration, JaeNeung University, Incheon 22573, Republic of Korea; bykim1@jeiu.ac.kr; Tel.: +82-32-890-7265

**Keywords:** weight-control medications, moderate-to-vigorous physical activity, physical activity, sex differences, obesity, health behaviour, cross-sectional study

## Abstract

**Background/Objectives:** The use of weight-control medications (WCMs) has increased rapidly, yet little is known about the physical activity (PA) behaviours of users. Therefore, the present study aimed to identify factors associated with moderate-to-vigorous physical activity (MVPA) among Korean WCM users and to examine sex-specific differences. **Methods:** This cross-sectional study analysed data from the 2024 Korea Community Health Survey (KCHS). A total of 6604 adults (men: *n* = 1209; women: *n* = 5395) using WCMs for weight control were included. Multivariable logistic regression, accounting for complex survey design weights, was conducted separately for men and women. **Results:** The weighted prevalence of MVPA was higher in men (32.3%) than in women (20.9%). Among men, being unmarried/divorced/widowed (OR = 1.52, 95% CI 1.12–2.07) was positively associated with MVPA, whereas being aged ≥60 years (OR = 0.48, 95% CI 0.28–0.83) was associated with lower odds. Among women, being aged 50–59 years (OR = 1.38, 95% CI 1.07–1.78) was associated with higher MVPA participation, whereas poor self-rated health (OR = 0.60, 95% CI 0.47–0.76) was associated with lower odds. **Conclusions:** Factors associated with MVPA among WCM users differed between men and women, suggesting that sex-specific approaches may be beneficial when promoting PA in this population. This study is limited by its cross-sectional design and reliance on self-reported data, which preclude causal inference.

## 1. Introduction

The management of obesity reached a historical turning point with the release of the global guideline on the use of glucagon-like peptide-1 (GLP-1) medicines in treating obesity by the World Health Organization (WHO) on 1 December 2025 [1]. In this context, the global use of weight-control medications (WCMs) has continued to increase substantially in recent years. In particular, GLP-1 receptor agonists—such as semaglutide, liraglutide, and tirzepatide—have become widely adopted as core pharmacological strategies for weight management. South Korea reflects this global trend; the domestic obesity pharmacotherapy market increased by 51.0% during the first half of 2025 compared with the previous year, reaching approximately KRW 270 billion [2]. The continued introduction of new agents is expected to further increase the use of pharmacological weight-management strategies. Beyond GLP-1, real-world weight control also involves non-prescription products and, in countries such as Korea, traditional herbal medicine; the population attempting pharmacological or herbal weight control is therefore broader than users of approved anti-obesity drugs alone.

Despite their effectiveness in promoting short-term weight loss, WCMs alone are often insufficient to sustain long-term metabolic health. Weight regain after discontinuation has been frequently reported, indicating that the behavioural and physiological drivers of obesity often persist after treatment cessation [3,4]. Lifestyle behaviours therefore remain a fundamental component of obesity management, and physical activity (PA), particularly at moderate-to-vigorous intensity, plays an important role in weight management: regular activity helps preserve lean muscle mass, maintain resting metabolic rate, and sustain the weight lost through WCM use [5]. Yet there is growing concern that increasing reliance on convenient pharmacological weight-control methods may lead individuals to place less emphasis on PA.

WCMs—and GLP-1 receptor agonists in particular—also produce broader physiological effects relevant to PA: by enhancing satiety and improving glycaemic control they reduce cardiometabolic risk, while the accompanying rapid weight loss can reduce lean muscle mass, an effect that can be attenuated by concurrent PA. For these reasons, PA is increasingly regarded not as an optional complement to pharmacotherapy but as an important determinant of the long-term effectiveness of WCM treatment [5]. Despite this, how WCMs influence users’ engagement in, and adherence to, regular PA remains poorly understood.

Substantial sex differences in PA participation have been consistently reported in population-based studies. This pattern has been documented both in global monitoring efforts led by the WHO and in large multi-country studies, with women generally reporting lower levels of PA than men [6]. For example, data from the 2020 U.S. National Health Interview Survey showed that 28.3% of men met combined aerobic and muscle-strengthening activity guidelines compared with 20.4% of women.

Numerous determinants of PA have been identified, including social support and marital status [7,8,9], socioeconomic factors such as education and employment [10,11,12], and health behaviours such as smoking, alcohol consumption, and sleep duration [13,14,15,16].

Body measures are also relevant: higher Body mass index (BMI) is associated with lower PA [17], whereas body-shape perception independently shapes exercise behaviour and often differs by sex [18,19,20,21]. Better self-rated health predicts greater PA [22], chronic conditions such as hypertension and diabetes may both motivate and hinder activity [23,24,25], and perceived stress may reduce PA [26,27].

The outcome of interest in studies of PA is frequently expressed as moderate-to-vigorous physical activity (MVPA), which reflects activity performed at an intensity sufficient to confer health benefits. International frameworks—including those of the World Health Organization and the Physical Activity Guidelines for Americans, together with comparable Korean national guidelines—consistently define MVPA as regular moderate- or vigorous-intensity activity meeting a minimum weekly threshold [28,29]. MVPA therefore provides a standardized and internationally comparable indicator of whether individuals reach health-enhancing levels of activity.

Although the clinical effectiveness of WCMs has been widely studied, most previous research has focused primarily on treatment outcomes such as weight reduction or body-composition changes when exercise is combined with pharmacological treatment [30,31]. In contrast, limited research has examined patterns of PA behaviour among individuals currently using WCMs. In particular, the factors associated with PA participation in this population—and whether these factors differ between men and women—remain insufficiently studied.

Addressing this gap is important: identifying which users are least active, and how the determinants of activity differ by sex, is a prerequisite for sex-specific strategies that help WCM users remain physically active and preserve the benefits of weight-control treatment. Therefore, using nationally representative data from the 2024 Korea Community Health Survey (KCHS), the present study aimed to (1) compare the weighted prevalence of MVPA between male and female WCM users; (2) identify sociodemographic, health-related, behavioural, and psychological factors associated with MVPA separately by sex; and (3) provide evidence that may inform the development of sex-specific strategies to promote PA among individuals using WCMs.

## 2. Materials and Methods

### 2.1. Study Design and Data Source

This study employed a cross-sectional design [32] using secondary data from the 2024 KCHS. The KCHS is a nationwide health survey conducted annually by the Korea Disease Control and Prevention Agency (KDCA) in collaboration with local public health centers. The survey aims to monitor the health status of residents and identify factors associated with major public health conditions. The survey uses a complex multistage sampling design, and the resulting data are representative of the adult population in South Korea [33].

**Figure 1:**
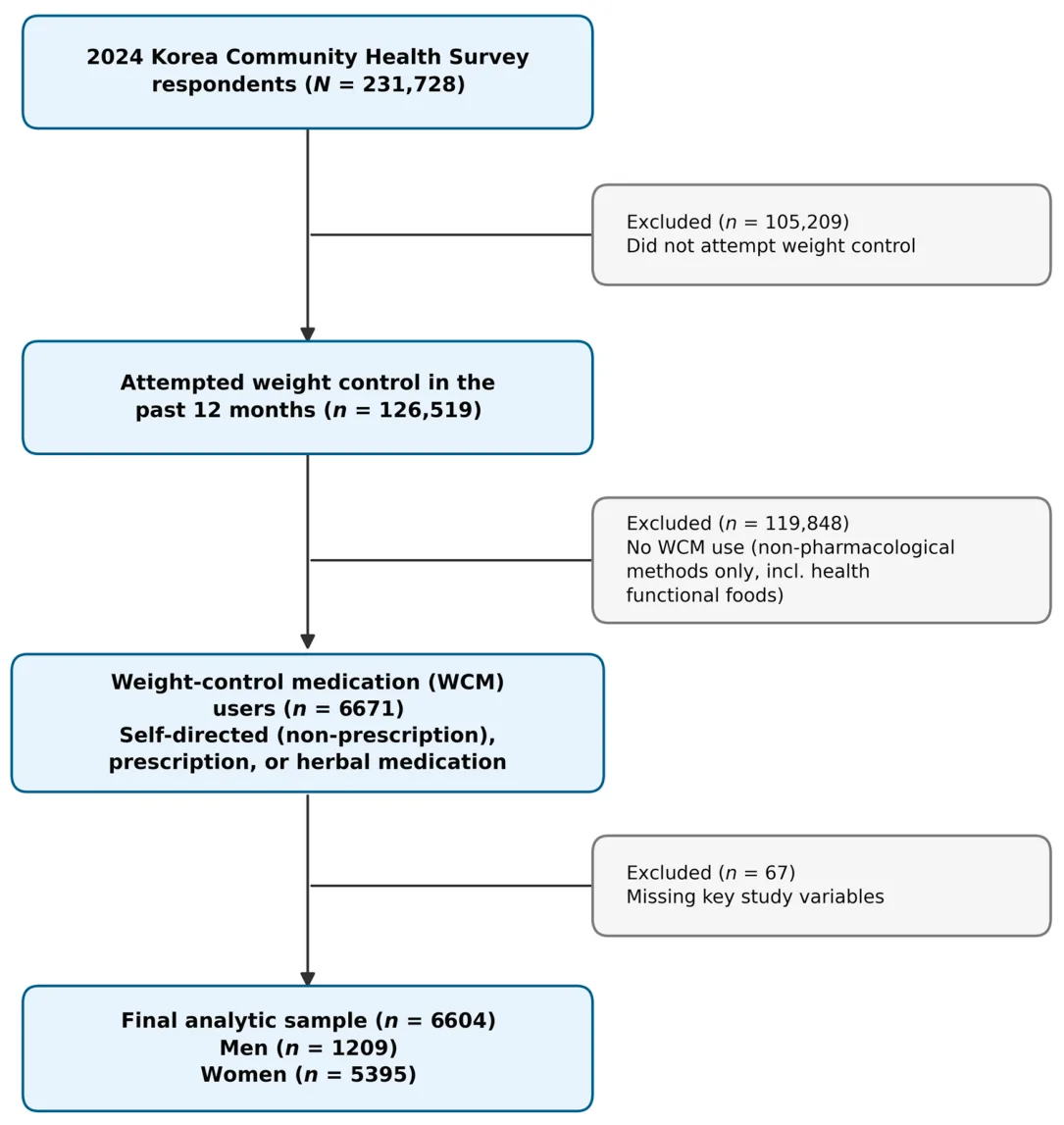
Flow diagram of participant selection. Weight-control medication (WCM) use was defined as self-reported use of self-directed (non-prescription) medication, physician-prescribed medication, or herbal medication for weight control. Non-pharmacological methods and health functional foods (dietary supplements) were not classified as WCMs and were excluded from the WCM definition.

The 2024 KCHS surveyed 231,728 community-dwelling adults. Participants were eligible if they reported attempting to lose or maintain body weight during the preceding 12 months; those for whom the weight-control methods item was not applicable (*n* = 105,209) were excluded, leaving 126,519 individuals who had attempted weight control. Of these, 119,848 individuals who reported using only non-pharmacological methods—including health functional foods—and no WCM were excluded, yielding 6671 WCM users. After further excluding 67 participants with missing values on any key study variable, the final analytic sample comprised 6604 participants (1209 men and 5395 women). The participant selection process is summarized in Figure 1.

In this study, WCMs were defined broadly to reflect real-world weight-control practice in Korea. WCM use was ascertained from a self-reported item in the KCHS that asked participants to report all methods used to lose or maintain body weight during the preceding 12 months. Participants were classified as WCM users if they reported using any of the following for weight control: (i) physician-prescribed weight-loss medication, (ii) non-prescription (over-the-counter or self-obtained) weight-loss medication, or (iii) herbal medicine (traditional Korean medicine). Health functional foods, which are regulated as dietary supplements rather than medicines, were not regarded as WCMs and were excluded. Because the survey recorded only the category of method used, the specific agent (including whether a GLP-1 receptor agonist was used), dose, and duration of treatment were not available.

The inclusion of herbal medicine reflects the distinctive dual structure of health care in Korea, where traditional Korean medicine operates as a licensed, insurance-covered system in parallel with conventional medicine. Its normalization is embedded in the survey instrument itself: the KCHS enumerates herbal medicine as a discrete, standalone response option for weight control, alongside prescription and non-prescription pharmacotherapy. A nationally administered survey would not measure herbal medicine as a separate weight-control modality unless its use were sufficiently common in the general population to warrant distinct measurement. Excluding herbal medicine would therefore systematically undercount the medication-using population—particularly women—and undermine the sex-specific focus of this study.

The data used in this study were obtained from the KDCA after formal approval of a data request. The data use agreement was completed, and the data request was applied for and approved on 16 June 2026. The approved dataset was used for the present analysis.

### 2.2. Measures

The primary outcome variable in this study was participation in MVPA. MVPA was defined according to national PA guidelines. Participants were classified as physically active if they reported engaging in vigorous-intensity PA (e.g., running, fast cycling, football, or heavy lifting) for at least 20 min on three or more days during the previous week, or moderate-intensity PA (e.g., brisk walking, slow swimming, doubles tennis, badminton, or table tennis) for at least 30 min on five or more days during the same period. Participants who met either criterion were classified as achieving the recommended level of MVPA.

Covariates were selected based on findings from previous studies examining determinants of PA and were grouped into several domains, including sociodemographic characteristics, health-related factors, health behaviours, and mental health indicators.

Sociodemographic characteristics included age, marital status, household income, educational attainment, and employment status. Age was categorized into five groups (20–29, 30–39, 40–49, 50–59, and ≥60 years). These age bands correspond to the age strata used for post-stratification weighting in the KCHS. Marital status was classified as married or unmarried/divorced/widowed. Household monthly income was divided into four categories (<1 million KRW, 1–3 million KRW, 3–5 million KRW, and ≥5 million KRW). These income brackets follow those used in the KCHS. Educational attainment was classified as ≤middle school, high school, or ≥college/university. Employment status was defined as being employed or unemployed, where employment referred to having performed at least one hour of paid work or at least 18 h of unpaid family work during the previous week.

Health-related variables included BMI, perceived body shape, self-rated health, and physician-diagnosed hypertension and diabetes. BMI was categorized into three groups: normal weight or underweight, overweight (25.0–29.9 kg/m^2^), and obese (≥30.0 kg/m^2^). These categories follow the World Health Organization international BMI classification [34]. The underweight and normal-weight categories were combined due to the small number of underweight participants. Perceived body shape was measured based on participants’ self-assessment and categorized as ≤slightly underweight, normal, slightly obese, or very obese. Self-rated health was assessed using a standard survey question and categorized as good, moderate, or poor. Participants were also asked whether they had ever been diagnosed with hypertension or diabetes by a physician, and responses were classified as yes or no.

Health behaviour variables included current smoking status, high-risk alcohol consumption, and weekday sleep duration. Current smoking was defined as currently smoking cigarettes at the time of the survey. High-risk drinking was defined as consuming seven or more standard drinks per occasion for men or five or more drinks for women at least twice per week. Weekday sleep duration was categorized into three groups: <7 h, 7 to <9 h, and ≥9 h per day [35].

Mental health status was assessed using self-reported perceived stress level. Participants reporting high or moderate stress were classified as having elevated stress, whereas those reporting low or no stress were classified as having low stress.

All variables used in this study were derived from self-reported responses collected through the KCHS.

### 2.3. Statistical Analysis

All statistical analyses were conducted using SAS version 9.4 (SAS Institute Inc., Cary, NC, USA). A significance level of α = 0.05 was applied for all statistical tests. Because the KCHS is based on a stratified, multistage probability sampling design, all analyses were weighted and incorporated the person-level sampling weight, the stratification variable, and the cluster (primary sampling unit) variable provided in the dataset. These weights were constructed and released by the KDCA: a household weight was first derived from the household extraction rate, the eligible-household rate, and the housing-type household ratio; it was multiplied by the individual response rate to obtain a person weight; and this person weight was then post-stratified to the resident-registration population of the survey year by sex and five age groups (19–29, 30–39, 40–49, 50–59, and ≥60 years), with extreme values trimmed using a raking-ratio procedure. In every analysis the person weight was applied through the WEIGHT statement, and the stratum and cluster variables through the STRATA and CLUSTER statements, of the SAS survey procedures (PROC SURVEYFREQ and PROC SURVEYLOGISTIC), with design-based variances estimated by Taylor series linearization. This weighting approach ensured that the estimates represented the target population of Korean adults using WCMs rather than the unweighted sample.

Weighted frequencies and proportions were calculated to describe participant characteristics separately for men and women. Because the primary outcome (MVPA) was binary and all covariates were categorical, no assumption of normality applied. Differences in the prevalence of MVPA across participant characteristics were examined using the Rao–Scott chi-square test, which adjusts for the complex survey design.

To identify factors independently associated with MVPA participation, multivariable logistic regression analyses were conducted separately for men and women, with all sociodemographic, health-related, behavioural, and mental-health covariates entered into the models simultaneously. Because WCM use was an eligibility criterion, it had no variance and could not be modeled as an interaction term with sex; men and women were therefore analysed in fully sex-stratified models, allowing the association of every covariate with MVPA to differ by sex. Because MVPA was a binary outcome, the odds ratio was used as the measure of effect size. The magnitude of each significant association was interpreted as small, medium, or large following established conventions for odds ratios, whereby odds ratios of approximately 1.68, 3.47, and 6.71 (or their reciprocals, 0.60, 0.29, and 0.15) denote small, medium, and large effects, respectively [36]. Results from the regression analyses are presented as adjusted odds ratios (aORs) with 95% confidence intervals, and a separate reference category was specified for each categorical predictor. Model adequacy was evaluated using the likelihood ratio F-statistic and Wald F-statistic, and model discrimination was evaluated using the percentage of concordant pairs. Multicollinearity among covariates was assessed using variance inflation factors (VIFs); all VIFs were below 2 (maximum 1.73), indicating no serious multicollinearity. To formally test whether the associations between covariates and MVPA differed by sex, an additional model including sex-by-covariate interaction terms was fitted on the full sample under the same complex-survey design, and the significance of each interaction was assessed using a design-based joint (Wald F) test. In addition to the multivariable models, unadjusted (crude) odds ratios were estimated for each covariate using separate univariable survey logistic regression models under the same complex-survey design and are reported in Supplementary Table S1. To examine the shape of the age–MVPA association, a test for linear trend across the ordered age categories and a design-based test for departure from linearity were additionally performed, separately for men and women.

## 3. Results

### 3.1. Sociodemographic, Health, and Behavioural Characteristics by Sex

Table 1 presents the baseline characteristics of the study population. Men and women differed significantly across most sociodemographic, health-related, and behavioural variables (all *p* < 0.05): compared with women, men were older, more often employed, and more frequently overweight or obese, and had higher prevalences of hypertension, diabetes, current smoking, and high-risk drinking. Educational attainment, self-rated health, and perceived stress did not differ significantly by sex. The weighted prevalence of MVPA was significantly higher among men (32.3%) than among women (20.9%) (*p* < 0.001).

### 3.2. MVPA Prevalence by Participant Characteristics

Supplementary Table S2 shows the prevalence of MVPA according to participant characteristics, stratified by sex. Among men, MVPA prevalence differed significantly by age, marital status, household income, education, employment, BMI, and perceived body shape, being highest in men aged 20–29 years (41.2%) and lower among those who perceived themselves as slightly obese (28.4%). Among women, it differed by educational attainment, perceived body shape, self-rated health, hypertension or diabetes, and sleep duration, and was lowest among women sleeping nine or more hours per day (11.0%). Full estimates are provided in Supplementary Table S2.

### 3.3. Multivariable Logistic Regression Analysis

**Figure 2:**
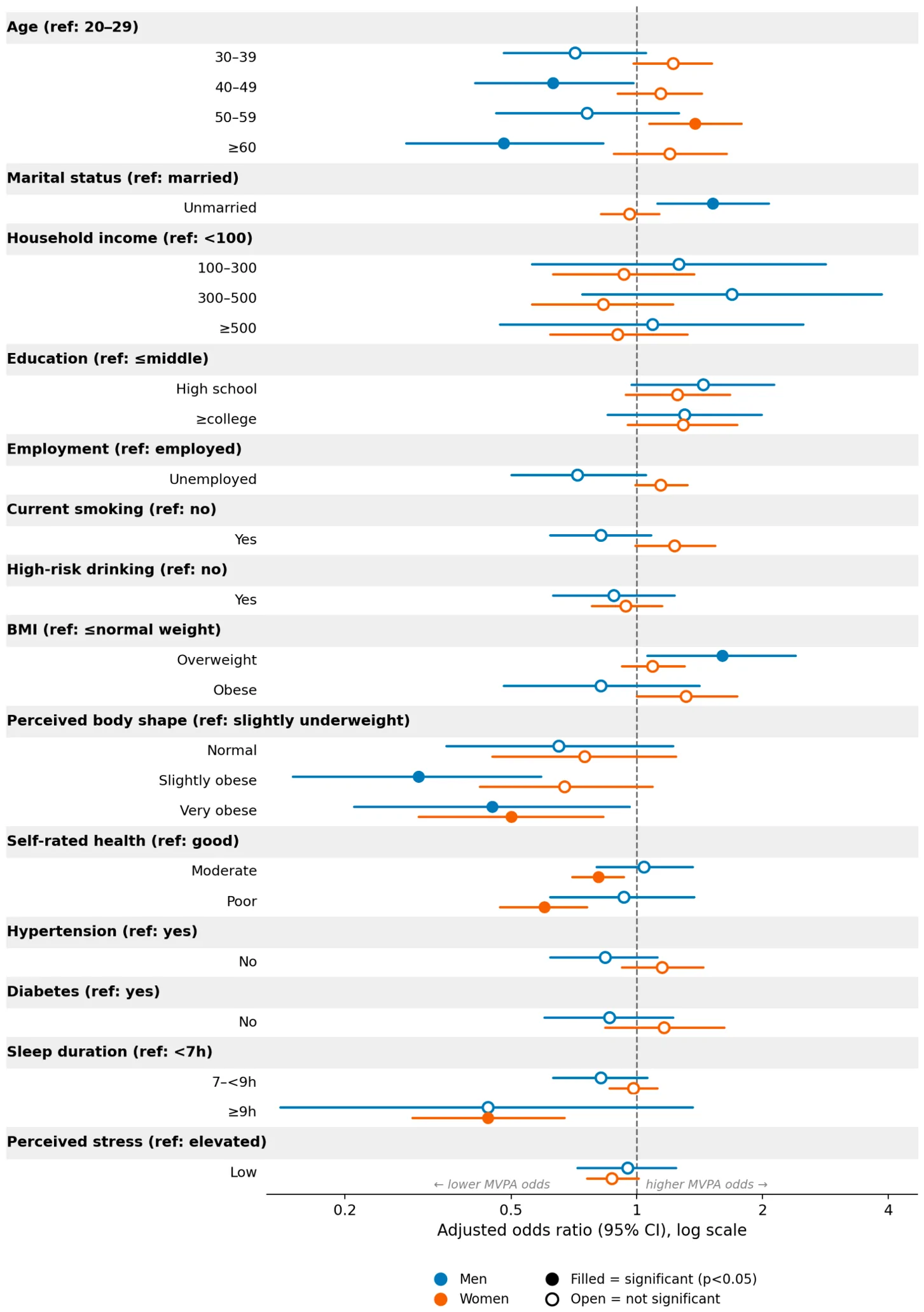
Forest plot of adjusted odds ratios (95% confidence intervals) for factors associated with moderate-to-vigorous physical activity (MVPA) among men and women. Filled circles denote statistically significant associations (*p* < 0.05) and open circles denote non-significant associations; each estimate is expressed relative to the reference category of the corresponding variable, and the dashed vertical line marks an odds ratio of 1.

Table 2 and Table 3 present the results of multivariable logistic regression analyses examining factors associated with MVPA separately for men and women. The corresponding adjusted odds ratios and 95% confidence intervals for both sexes are additionally visualized in Figure 2. Formal tests of sex-by-covariate interaction confirmed that several associations differed significantly between men and women, including those for BMI (*p* = 0.0004), perceived body shape (*p* = 0.0005), marital status (*p* = 0.011), employment (*p* = 0.026), current smoking (*p* = 0.026), and household income (*p* = 0.027), whereas the sex-by-age interaction was of borderline significance (*p* = 0.051). Unadjusted (crude) odds ratios for all covariates are provided in Supplementary Table S1.

Among men, age was negatively associated with MVPA participation. Compared with men aged 20–29 years, men aged 40–49 years had lower odds of meeting MVPA guidelines (OR = 0.63, 95% CI: 0.41–0.98), and men aged 60 years or older also had reduced odds of MVPA (OR = 0.48, 95% CI: 0.28–0.83). Marital status was significantly associated with MVPA; men who were unmarried, divorced, or widowed had higher odds of engaging in MVPA than married men (OR = 1.52, 95% CI: 1.12–2.07). BMI was also associated with MVPA, with overweight men showing higher odds of MVPA participation compared with those with normal weight (OR = 1.60, 95% CI: 1.06–2.40). In addition, perceived body shape was significantly associated with MVPA. Compared with men who perceived themselves as slightly underweight, those who perceived themselves as slightly obese had lower odds of MVPA (OR = 0.30, 95% CI: 0.15–0.59), and those who perceived themselves as very obese also had lower odds (OR = 0.45, 95% CI: 0.21–0.96).

Among women, different factors were associated with MVPA participation. Women aged 50–59 years had higher odds of engaging in MVPA compared with those aged 20–29 years (OR = 1.38, 95% CI: 1.07–1.78). Perceived body shape was also associated with MVPA; compared with women who perceived themselves as slightly underweight, women who perceived themselves as very obese had lower odds of meeting MVPA guidelines (OR = 0.50, 95% CI: 0.30–0.83). Self-rated health showed a graded association with MVPA. Compared with women reporting good health, women reporting moderate health had lower odds of MVPA (OR = 0.81, 95% CI: 0.70–0.93), and those reporting poor health had further reduced odds (OR = 0.60, 95% CI: 0.47–0.76). Sleep duration was also associated with MVPA, with women who reported sleeping nine or more hours per day having lower odds of MVPA compared with those sleeping fewer than seven hours per day (OR = 0.44, 95% CI: 0.29–0.67). When interpreted by these conventions, the significant associations ranged from small to medium in magnitude. The largest was for perceived body shape in men (OR = 0.30, a medium effect); long sleep duration in women (OR = 0.44) and older age (≥60 years) in men (OR = 0.48) fell between small and medium, whereas marital status, individual age groups, and self-rated health corresponded to small effects.

## 4. Discussion

This study examined sex-specific determinants of MVPA among Korean adults currently using WCMs. The results indicate that the prevalence of MVPA is higher among men than among women, and that the factors associated with MVPA participation differ between the two groups. These sex differences in the determinants of MVPA were further supported by formal sex-by-covariate interaction tests, which reached statistical significance for several factors.

### 4.1. Sex Differences in the Prevalence of MVPA

The prevalence of MVPA observed in this study was 32.3% among men and 20.9% among women. This pattern is consistent with previous population-based studies reporting higher levels of PA among men than among women. For example, a large international meta-analysis reported that approximately 23.5% of men and 17.4% of women met recommended PA guidelines across multiple countries. The difference observed in the present study appears somewhat larger than those reported in general population studies.

### 4.2. Sex-Specific Determinants of MVPA

Age was associated with MVPA participation in both men and women, although the pattern of association appeared to differ between the sexes, with the sex-by-age interaction reaching only borderline significance (*p* = 0.051). Specifically, although the overall age effect did not reach statistical significance in formal trend or departure-from-linearity tests, specific age groups differed: men aged 40–49 and ≥60 years had lower odds than those aged 20–29, whereas women aged 50–59 had higher odds. These age patterns are therefore presented as category-specific associations rather than as an overall linear or non-linear trend. Among men, the odds of meeting MVPA guidelines were lower in the older age groups, particularly among those aged 60 years or older. This is consistent with previous research indicating that PA tends to be lower at older ages, partly due to changes in physical capacity, health status, and lifestyle patterns [17,37]. In contrast, women aged 50–59 years showed higher odds of engaging in MVPA compared with women in their twenties. Similar patterns have been reported in studies suggesting that middle-aged women may increase participation in health-related behaviours as family responsibilities decrease and health awareness increases [12,38].

Perceived body shape was significantly associated with MVPA participation in both men and women. However, the pattern of association differed between the sexes. Among men, compared with those who perceived themselves as slightly underweight, those who perceived themselves as slightly obese or very obese had lower odds of meeting MVPA guidelines. In contrast, among women, only those who perceived themselves as very obese showed significantly lower MVPA participation compared with those who perceived themselves as slightly underweight. These findings suggest that body image perception may influence PA behaviours differently between men and women. Previous studies have indicated that men’s PA may be more closely related to functional or performance-related perceptions, whereas women’s behaviours may be more strongly influenced by appearance-related or sociocultural factors [20,21].

Among men, marital status was associated with MVPA participation, with unmarried men showing higher odds of meeting MVPA guidelines than married men. Previous studies have suggested that family responsibilities and time constraints associated with married life may influence participation in leisure-time PA [39]. BMI was also associated with MVPA among men, with overweight men showing higher odds of MVPA participation compared with men of normal weight.

Among women, multiple factors were associated with MVPA participation. Self-rated health showed a graded relationship with MVPA, with lower levels of PA observed among women reporting poorer health status. Similar associations have been widely reported in previous studies, where individuals with poorer perceived health are less likely to engage in PA [40]. Sleep duration was also associated with MVPA among women in this study. Women reporting nine or more hours of sleep per day had lower odds of meeting MVPA guidelines compared with those with shorter sleep durations. Although the mechanisms underlying this relationship remain unclear, longer sleep duration has been linked in some studies to lower energy levels, underlying health conditions, or reduced daily activity patterns [16,41].

The present findings should be interpreted alongside the emerging body of research on PA among users of WCMs. Randomized controlled trials have shown that combining structured exercise with GLP-1 receptor agonist therapy improves the maintenance of weight loss and better preserves lean body mass than pharmacotherapy alone [42], and that such combined approaches produce greater reductions in metabolic-syndrome severity and abdominal obesity than either strategy in isolation [43]. These trial findings reinforce reviews that emphasize the complementary role of PA during pharmacological weight management [5,31]. The present study extends this literature by profiling MVPA among real-world WCM users and, importantly, by identifying sex-specific subgroups—older men and those with a distorted body-shape perception, and women with poor self-rated health or long sleep duration—who are least likely to achieve recommended activity levels and may therefore gain the least from the long-term metabolic benefits of combined treatment.

These associations should also be interpreted within their socioeconomic context. Education, household income, and employment status shape both opportunities and constraints for PA through pathways such as available leisure time, occupational demands, financial access to exercise facilities, and health literacy [10,11,12].

Taken together, the findings suggest that factors associated with PA among individuals using WCMs may differ between men and women. These differences highlight the potential importance of considering sex-specific factors when designing interventions to promote PA in this population.

This study has several limitations. First, because the study used cross-sectional data from the KCHS, causal relationships between the identified factors and PA cannot be established. Second, WCM use was assessed as a single binary variable in the survey, and information on specific medication types, dosage, or duration of treatment was not available. Different medications may have different physiological effects that could influence PA behaviour. Moreover, WCM use was defined broadly, combining prescription, non-prescription, and herbal preparations into a single category, so the findings pertain to the broad population using pharmacological or herbal weight control rather than to any specific drug class such as GLP-1 receptor agonists. Because the study included only WCM users and no medication-free comparison group, it also cannot establish whether medication use itself influences PA. Third, the number of male participants in the study was smaller than the number of female participants, which may have reduced statistical power for detecting associations among men. Fourth, PA and all other study variables were assessed using self-reported data, which may be subject to recall and social-desirability biases and to misclassification. Finally, some environmental and psychological factors that may influence PA, such as neighborhood environment, access to exercise facilities, and intrinsic motivation, were not available in the survey dataset.

### 4.3. Strengths and Future Research

This study has several notable strengths. It is based on nationally representative data from a large-scale community health survey and incorporated complex survey weights, stratification, and clustering, allowing the findings to be generalized to the Korean adult population using WCMs. Furthermore, this study is one of the few to examine the sex-specific determinants of PA among WCM users—a fast-growing yet understudied population that includes users of the rapidly adopted GLP-1 receptor agonists. The separate modelling of men and women also enabled the identification of distinct behavioural profiles that could be obscured in pooled analyses.

Notably, most WCM users did not meet MVPA recommendations (67.7% of men and 79.1% of women), underscoring that PA should not be neglected even among individuals using pharmacological or herbal weight-control methods. From a practical perspective, the sex-specific patterns identified in this study can inform the design of targeted PA interventions for individuals using WCMs. For men, intervention efforts may prioritize older individuals and those who perceive themselves as obese, as these groups showed lower levels of PA. For women, greater attention may be directed toward those reporting poor self-rated health or excessively long sleep duration, who were less likely to meet MVPA recommendations. Integrating structured PA counselling into pharmacological weight-management programmes may help preserve lean body mass, sustain metabolic benefits, and reduce the risk of weight regain following treatment discontinuation.

Beyond documenting sex-specific patterns, these findings carry several practical and policy implications. In clinical practice, brief assessment and counselling of PA could be integrated into the routine follow-up of individuals receiving weight-control treatment, with particular attention to the subgroups least likely to be active—older men and men who perceive themselves as obese, and women reporting poor self-rated health or long sleep duration. At the level of clinical guidance, recommendations for WCM could more explicitly endorse concurrent PA and incorporate the monitoring of activity levels, thereby reinforcing the complementary role of lifestyle behaviour during pharmacological weight management. Finally, the divergent correlates observed in men and women suggest that behavioural interventions may prove more effective when tailored by sex—for instance, emphasising functional and performance-related goals for men and health- and appearance-related motivations for women. Collectively, these measures could help ensure that pharmacologically induced weight loss is accompanied by the PA needed to preserve lean mass and to sustain its long-term metabolic benefits.

Future research should build on these findings in several directions. Longitudinal studies are needed to clarify the temporal and causal relationships between the identified factors and PA participation. In addition, studies incorporating detailed clinical information—such as medication type, dosage, and treatment duration—together with objectively measured PA would strengthen and extend the present findings. Intervention studies evaluating sex-specific strategies to promote PA among WCM users are also warranted to translate these observations into effective clinical and public health practice.

## 5. Conclusions

This study examined determinants of MVPA among Korean adults using WCMs based on nationally representative survey data. The prevalence of MVPA is higher among men than among women, and several determinants of PA differ between the two groups.

Among men, older age and perceiving oneself as slightly obese are associated with lower MVPA participation, whereas being unmarried and having an overweight BMI are associated with higher levels of PA. Among women, MVPA participation is positively associated with being aged 50–59 years and negatively associated with perceiving oneself as very obese, reporting poorer self-rated health, and sleeping longer hours.

These findings suggest that determinants of PA among individuals using WCMs may differ between men and women. Understanding these differences may help inform the development of targeted strategies to promote PA among individuals undergoing pharmacological treatment for weight control. Future studies using longitudinal designs and incorporating detailed information on medication type and treatment characteristics may further clarify the behavioural and clinical implications of WCM use.

## Figures and Tables

**Table 1:** Socio-demographic characteristics and health behaviours by sex.

Variable	Category	Male (*n* = 1209) N	%	Female (*n* = 5395) N	%	*p*
Age	20–29	149	16.1	777	18.9	<0.0001
	30–39	315	31.7	1192	24.8	
	40–49	307	25.2	1453	27.9	
	50–59	182	14.2	1138	18.4	
	≥60	256	12.7	835	9.9	
Marriage status	Yes	745	57.7	3538	63.0	0.0006
	No	464	42.3	1857	37.0	
Household monthly income	<100	39	1.9	173	2.4	0.0005
	100–300	209	13.0	1086	17.1	
	300–500	323	26.7	1333	23.8	
	≥500	638	58.3	2803	56.6	
Education level	≤Middle	135	6.8	658	7.6	0.0838
	High school	443	36.1	2145	38.2	
	≥college/university	631	57.1	2592	54.1	
Employment status	Yes	1006	83.8	3843	70.6	<0.0001
	No	203	16.2	1552	29.4	
Current smoking	Yes	416	36.2	471	9.4	<0.0001
status	No	793	63.8	4924	90.6	
High-risk alcohol	Yes	264	21.5	667	13.0	<0.0001
consumption	No	945	78.5	4728	87.0	
BMI	≤normal weight	255	20.2	2919	56.8	<0.0001
	Overweight	605	49.8	1878	33.1	
	Obese	349	30.0	598	10.1	
Perceived	Slightly underweight	45	2.6	102	1.9	<0.0001
body shape	Normal	236	18.2	1182	23.2	
	slightly obese	579	47.4	2793	51.6	
	very obese	349	31.8	1318	23.3	
Self-rated health	Good	482	41.5	1980	39.3	0.2817
	Moderate	557	46.0	2661	48.2	
	Poor	170	12.5	754	12.5	
Hypertension	Yes	401	28.8	1021	14.5	<0.0001
	No	808	71.2	4374	85.5	
Diabetes	Yes	194	13.4	396	5.7	<0.0001
	No	1015	86.6	4999	94.3	
Sleep duration	<7	604	53.0	2689	50.3	0.0491
	7 to <9	569	44.1	2497	45.6	
	≥9	36	2.8	209	4.1	
Stress level	elevated stress	382	35.0	1871	37.0	0.1895
	low stress	827	65.0	3524	63.0	
MVPA	Yes	386	32.3	1142	20.9	<0.0001
	No	823	67.7	4253	79.1	

**Table 2:** Multivariable logistic regression analysis of factors associated with Moderate-to-vigorous Physical activity (MVPA) in men.

Variable (Reference Group)	Category	Odds Ratio	OR 95% CI		*p*
Men (*n* = 1209)			Lower	Upper	
Age (20–29)	30–39	0.71	0.48	1.05	0.086
	40–49	0.63	0.41	0.98	<0.05
	50–59	0.76	0.46	1.26	0.289
	≥60	0.48	0.28	0.83	<0.05
Marriage status (Yes)	No	1.52	1.12	2.07	<0.05
Household monthly income (<100)	100–300	1.26	0.56	2.83	0.568
	300–500	1.69	0.74	3.86	0.214
	≥500	1.09	0.47	2.50	0.843
Education level (≤Middle)	High school	1.44	0.97	2.13	0.068
	≥college/university	1.30	0.85	1.99	0.226
Employment status (Yes)	No	0.72	0.50	1.05	0.084
Current smoking status (No)	Yes	0.82	0.62	1.08	0.156
High-risk alcohol consumption (No)	Yes	0.88	0.63	1.23	0.447
BMI (≤normal weight)	overweight	1.60	1.06	2.40	<0.05
	obese	0.82	0.48	1.41	0.476
Perceived body shape	normal	0.65	0.35	1.22	0.179
(Slightly underweight)	slightly obese	0.30	0.15	0.59	<0.05
	very obese	0.45	0.21	0.96	<0.05
Self-rated health (Good)	moderate	1.04	0.80	1.36	0.767
	Poor	0.93	0.62	1.37	0.696
Hypertension (Yes)	No	0.84	0.62	1.12	0.234
Diabetes (Yes)	No	0.86	0.60	1.22	0.392
Sleep duration (<7)	7 to <9	0.82	0.63	1.06	0.132
	≥9	0.44	0.14	1.36	0.153
Stress level (elevated stress)	low stress	0.95	0.72	1.24	0.685

**Table 3:** Multivariable logistic regression analysis of factors associated with Moderate-to-vigorous Physical activity (MVPA) in women.

Variable (Reference Group)	Category	Odds Ratio	OR 95% CI		*p*
Women (*n* = 5395)			Lower	Upper	
Age (20–29)	30–39	1.22	0.98	1.51	0.081
	40–49	1.14	0.90	1.43	0.278
	50–59	1.38	1.07	1.78	<0.05
	≥60	1.20	0.88	1.64	0.248
Marriage status (Yes)	No	0.96	0.82	1.13	0.617
Household monthly income (<100)	100–300	0.93	0.63	1.37	0.699
	300–500	0.83	0.56	1.22	0.346
	≥500	0.90	0.62	1.32	0.599
Education level (≤Middle)	High school	1.25	0.94	1.67	0.127
	≥college/university	1.29	0.95	1.74	0.106
Employment status (Yes)	No	1.14	0.99	1.32	0.076
Current smoking status (No)	Yes	1.23	0.99	1.54	0.064
High-risk alcohol consumption (No)	Yes	0.94	0.78	1.15	0.557
BMI (≤normal weight)	overweight	1.09	0.92	1.30	0.310
	obese	1.31	1.00	1.74	0.055
Perceived body shape	normal	0.75	0.45	1.24	0.258
(Slightly underweight)	slightly obese	0.67	0.42	1.09	0.104
	very obese	0.50	0.30	0.83	<0.05
Self-rated health (Good)	moderate	0.81	0.70	0.93	<0.05
	Poor	0.60	0.47	0.76	<0.0001
Hypertension (Yes)	No	1.15	0.92	1.44	0.213
Diabetes (Yes)	No	1.16	0.84	1.62	0.370
Sleep duration (<7)	7 to <9	0.98	0.86	1.12	0.773
	≥9	0.44	0.29	0.67	<0.05
Stress level (elevated stress)	low stress	0.87	0.76	1.01	0.063

## Data Availability

The data used in this study are available in a publicly accessible repository, the Korea Community Health Survey (KCHS) https://chs.kdca.go.kr/chs/rawDta/rawDtaProvdMain.do (accessed on 13 August 2026), approved under National Statistics Approval No. 117075.

## Supplementary Materials

The following supporting information can be downloaded at https://www.mdpi.com/article/10.3390/healthcare14162570/s1, Table S1: Unadjusted (crude) odds ratios for factors associated with moderate-to-vigorous physical activity (MVPA), by sex. Table S2: Prevalence of moderate-to-vigorous physical activity (MVPA) by general characteristics, by sex.

## Abbreviations

The following abbreviations are used in this manuscript:

KCHS	Korea Community Health Survey
KDCA	Korea Disease Control and Prevention Agency
MVPA	Moderate-to-vigorous Physical activity
GLP-1	Glucagon-like peptide-1
WCMs	Weight-control medications
PA	Physical activity
BMI	Body mass index
