# Supplementary material for: Sex-Specific Determinants of Physical Activity Among Korean Adults Using Weight-Control Medications: A Cross-Sectional Study
Source: Healthcare (Basel). 2026 Aug 17;14(16):2570. doi: 10.3390/healthcare14162570 (PMC13512236; doi:10.3390/healthcare14162570)
Supplement: Supplementary file 1 [file healthcare-14-02570-s001.zip › healthcare-4464267-supplementary.pdf]

Supplementary Materials

Sex-Specific Determinants of Physical Activity among Korean Adults Using Weight-Control Medications: A Cross-Sectional Study

Bo-Young Kim

Table S1. Unadjusted (crude) odds ratios for factors associated with moderate-to-vigorous physical activity (MVPA), by sex.

Characteristic	Men (*n* = 1209)		Women (*n* = 5395)	
	OR (95% CI)	*p*	OR (95% CI)	*p*
**Age group (ref: 20–29 years)**				
30–39 years	0.72 (0.49–1.05)	0.090	1.15 (0.95–1.40)	0.163
40–49 years	0.55 (0.37–0.81)	0.003	1.08 (0.88–1.32)	0.465
50–59 years	0.72 (0.46–1.13)	0.157	1.23 (0.99–1.52)	0.068
≥60 years	0.49 (0.34–0.73)	<0.001	0.92 (0.74–1.15)	0.479
**Marital status (ref: married)**				
Unmarried/divorced/widowed	1.65 (1.29–2.10)	<0.001	0.94 (0.82–1.06)	0.302
**Household monthly income (ref: <1 million KRW)**				
1–3 million KRW	1.51 (0.76–2.99)	0.243	0.97 (0.67–1.42)	0.884
3–5 million KRW	2.05 (1.04–4.06)	0.039	0.93 (0.64–1.36)	0.718
≥5 million KRW	1.34 (0.69–2.58)	0.386	1.04 (0.72–1.48)	0.852
**Education (ref: ≤middle school)**				
High school	1.56 (1.10–2.22)	0.014	1.36 (1.07–1.72)	0.011
≥College/university	1.27 (0.91–1.77)	0.163	1.40 (1.10–1.76)	0.005
**Employment (ref: employed)**				
Unemployed	0.78 (0.55–1.09)	0.148	1.04 (0.91–1.20)	0.554
**Current smoking (ref: no)**				
Yes	0.92 (0.71–1.18)	0.513	1.10 (0.91–1.33)	0.342
**High-risk alcohol use (ref: no)**				
Yes	0.88 (0.65–1.18)	0.385	0.95 (0.79–1.13)	0.538
**Body mass index (ref: ≤normal weight)**				
Overweight	1.05 (0.77–1.42)	0.767	0.89 (0.77–1.03)	0.122
Obese	0.75 (0.55–1.04)	0.086	0.84 (0.67–1.06)	0.138
**Perceived body shape (ref: slightly underweight)**				
Normal	0.79 (0.40–1.55)	0.490	0.81 (0.49–1.34)	0.403
Slightly obese	0.46 (0.24–0.89)	0.021	0.75 (0.47–1.19)	0.221
Very obese	0.56 (0.29–1.08)	0.081	0.57 (0.36–0.92)	0.021
**Self-rated health (ref: good)**				
Moderate	0.91 (0.70–1.17)	0.445	0.82 (0.72–0.94)	0.005
Poor	0.82 (0.57–1.19)	0.290	0.60 (0.47–0.76)	<0.001
**Hypertension (ref: yes)**				
No	1.09 (0.85–1.40)	0.498	1.25 (1.03–1.51)	0.026
**Diabetes (ref: yes)**				
No	0.94 (0.68–1.30)	0.721	1.35 (1.01–1.82)	0.045

**Sleep duration (ref: <7 h)**				
7 to <9 h	0.87 (0.68–1.11)	0.270	1.00 (0.88–1.13)	0.936
≥9 h	0.50 (0.17–1.49)	0.214	0.45 (0.30–0.68)	<0.001
**Perceived stress (ref: elevated)**				
Low	1.02 (0.79–1.31)	0.889	0.96 (0.84–1.10)	0.522

OR, odds ratio; CI, confidence interval. Odds ratios were estimated from separate univariate (unadjusted) survey logistic regression models for each characteristic, using domain (subpopulation) analysis that retained the full complex survey design (sampling weights, strata, and clusters), with variances estimated by Taylor series linearization. The reference category for each characteristic is indicated in parentheses.

Table S2. Prevalence of moderate-to-vigorous physical activity (MVPA) by general characteristics, by sex.

**Variable**	**Category**	**Male (*n*=1209)** Weighted % of physical activity (PA)	**P**	**Female (*n*=5395)** Weighted % of physical activity (PA)	**P**
Age	20-29	41.2	< .0001	19.5	0.1057
	30-39	33.6		21.8	
	40-49	27.7		20.7	
	50-59	33.7		22.9	
	≥60	25.7		18.3	
Marriage status	Yes	27.7	< .0001	21.3	0.2873
	No	38.7		20.2	
Household monthly income	<100	23.9	< .0001	20.9	0.5392
	100-300	32.1		20.5	
	300-500	39.2		19.8	
	≥500	29.6		21.5	
Education level	≤Middle	26.2	0.0018	16.4	0.0291
	High school	35.6		21.0	
	≥college/university	31.0		21.5	
employment status	Yes	33.2	0.0040	20.7	0.5480
	No	27.9		21.4	
current smoking	Yes	31.2	0.3508	22.4	0.3225
Status	No	33.0		20.8	

high-risk alcohol	Yes	30.1	0.1653	20.1	0.5287
Consumption	No	33.0		21.0	
BMI	≤normal weight	33.6	0.0003	21.8	0.1458
	Overweight	34.7		20.0	
	Obese	27.6		19.0	
Perceived	Slightly underweight	46.3	< .0001	26.8	0.0022
body shape	Normal	40.5		22.8	
	slightly obese	28.4		21.5	
	very obese	32.4		17.3	
self-rated health	Good	33.9	0.1834	23.6	< .0001
	Moderate	31.7		20.2	
	Poor	29.6		15.5	
hypertension	Yes	31.0	0.2882	18.0	0.0221
	No	32.9		21.4	
Diabetes	Yes	33.5	0.5464	16.6	0.0390
	No	32.2		21.2	
sleep duration	<7	34.1	0.1040	21.4	0.0003
	7 to <9	31.0		21.3	
	≥9	20.6		11.0	
stress level	elevated stress	32.1	0.8372	21.4	0.5147
	low stress	32.5		20.7	
